# Phytochemical Composition and Bioactivities of Aqueous Extract of Rambutan (*Nephelium lappaceum* L. cv. Rong Rian) Peel

**DOI:** 10.3390/antiox11050956

**Published:** 2022-05-12

**Authors:** Husanai Jantapaso, Pimonsri Mittraparp-arthorn

**Affiliations:** 1 Division of Biological Science, Faculty of Science, Prince of Songkla University, Hat Yai, Songkhla 90110, Thailand; dew22husanai@gmail.com; 2 Molecular Evolution and Computational Biology Research Unit, Faculty of Science, Prince of Songkla University, Hat Yai, Songkhla 90110, Thailand

**Keywords:** antioxidant, antibacterial, bioactive compounds, GC-MS, FT-IR, rambutan

## Abstract

Thailand is one of the leading exporting countries of rambutan and rambutan peels are considered as a biological waste. In this study, rambutan (*Nephelium lappaceum* L. cv. Rong Rian) peel extracts (RPE) obtained by water extraction were analyzed for their phytochemical composition, antibacterial and antioxidant activities, and cytotoxicity. The bioactive compounds in RPE identified by GC-MS were mome inositol (35.99 mg/g), catechol (29.37 mg/g), 5-hydroxymethylfurfural (5.69 mg/g), 2-pentenal, (E)-(5.22 mg/g), acetic acid (3.69 mg/g), 1,2,3-propanetriol (3.67 mg/g), 2-furan-carboxaldehyde (2.66 mg/g), and other compounds. FT-IR analysis confirmed the presence of alcohol and phenol in the extract. Antibacterial activities of RPE against food pathogenic and spoilage bacteria showed that RPE could inhibited *Bacillus subtilis*, *B*. *cereus*, *Staphylococcus aureus*, *Vibrio cholerae*, *V. parahaemolyticus*, *Pseudomonas aeruginosa*, and *P. fluorescens*, with MIC values ranging between 1024 and 8192 µg/mL. The extract also showed antioxidant properties, as determined by DPPH and ABTS assays. The cytotoxicity analysis after 72 h of treatment showed the IC_50_ values at 194.97 ± 4.87, 205.92 ± 2.55, and 94.11 ± 1.33 µg/mL for L929, Vero, and MCF-7 cell lines, respectively. Therefore, this study provided a basis of knowledge of rambutan peels as an excellent source of natural bioactive compounds for various applications.

## 1. Introduction

Rambutan (*Nephelium lappaceum* L.) is an economically important tropical fruit widely cultivated in south-east Asia and the cultivar “Rong Rian” or Nasan School Rambutan has long been known as the most popular and delicious rambutan in Thailand. Thailand is one of the world’s leading producers and exporters of the rambutan fruit and a variety of rambutan products, especially canned rambutan in syrup. Thus, large amounts of rambutan peels were uselessly discarded and considered as biological wastes [1,2]. Previous studies have reported that peels of rambutan contain phytochemical compounds including flavonoids, saponin, tannins, geraniin, ellagic acid, and corilagin that act as a potential of antioxidant and antibacterial activities [3,4]. Moreover, the antidiabetic and antihypercholesterolemic activities of rambutan peel extract (RPE) has been described [5]. The amount and variety of phytochemical compounds may differ depending on several factors, such as the region or country of cultivation, geographical climate, and soil condition [6]. Therefore, investigations into the phytochemical compounds and biological activities of rambutan peels are still necessary for their potential applications.

Food safety is a major health concern. Unsafe food may be contaminated with microorganisms and/or chemical substances. Some strains of bacteria can cause food spoilages and food poisoning diseases in human, e.g., *Bacillus* spp., *Clostridium perfringens*, *Staphylococcus aureus*, *Vibrio* spp., *Escherichia coli*, *Pseudomonas* spp., and *Salmonella* sp. [7]. To reduce or eliminate food-associated pathogens, chemicals or antibiotics are used, and these may damage the health of workers, environments, and other organisms [8]. The discovery of bioactive compounds from plant, especially agricultural wastes, as the alternatives to chemicals and antibiotics is interesting because various parts of plants contain bioactive compounds known as phytochemicals that exhibit the antimicrobial activity, antioxidant activity, and other activities that benefit human [9]. Rambutan fruit peels are considered as wastes and contain such powerful anti-bacteria and antioxidants. In this regard, many studies reported about the antibacterial activity of rambutan peel extract against *S. aureus*, *S. epidermidis*, *Listeria monocytogenes*, *E. coli*, *V. campbellii, V. cholerae, V. parahaemolyticus, V. anguillarum, P. aeruginosa, S. enteritidis,* and *Enterococcus faecalis* [5,10]. Antioxidants refer to the compounds that act as the reducing agents of free radicals which are harmful to the human body. It has been reported that free radicals contributed to aging, heart diseases, cancer, and other diseases [11]. In rambutan peel, phenolic and flavonoid compounds (i.e., ellagitannins, phenolic acid, quercetin, and gallotannin) were related to its antioxidant activity. In addition, the presence of vitamin compounds in the peel of rambutan is also involved in the antioxidant activity [12,13]. Thus, the aims of this study are to extract and identify the bioactive compounds from peels of Nasan School Rambutan and investigate its antibacterial, antioxidant, and cytotoxic activities.

## 2. Materials and Methods

### 2.1. Bacterial Strains

Food pathogenic and spoilage bacteria used in this study were *B. subtilis* PSU4655, *B. cereus* PSU3874, *S. aureus* ATCC25923, *V. cholerae* PSU6072, *V. parahaemolyticus* ATCC17802, *Salmonella* sp. PSU411, *E. coli* ATCC25922, *Pseudomonas aeruginosa* ATCC27853, and *P. fluorescens* PSU6074. All isolates were recovered from the microbial culture collection, Division of Biological Science, Prince of Songkla University, and were preserved in 20% glycerol at −80 °C.

### 2.2. Chemicals

The reagents used for antioxidant activity assay were 2,2-diphenyl-1-picrylhydrazyl (DPPH), 2,2′-azino-bis-(3-ethylbenzothiazoline-6-sulfonic acid (ABTS), 6-hydroxy-2,5,7,8-tetramethylchroman-2-carboxylic acid (Trolox), and potassium persulfate (K_2_S_2_O_8_), purchased from Sigma Aldrich (Darmstadt, Germany).

### 2.3. Preparation of Dried Rambutan Peels

Nasan School Rambutan (Ngo Rong Rean) used in this study were obtained directly from farm in Surat Thani province, southern Thailand, during fruiting season (July–September). Fresh rambutan peels were washed under running tap water and dried by hot air oven at 40 °C for 3 days or until dried. The dried rambutan peels were kept in a dry condition in the dark at room temperature until extraction.

### 2.4. Extraction of Rambutan Peel Extract (RPE) by Water Extraction

Dried rambutan peels were saturated in water using the method described by Hernandez et al. [7] with some modifications. Briefly, 100 g of dried rambutan peels was soaked in 500 mL of hot distilled water at 60 °C in ratio 1:5. The mixture was placed in a heating oven at 60 °C for 30 min. Before concentration of the extract, the peels were removed by filtrating through double layers of muslin. The solution was concentrated by freeze dryer (Martin Christ, Osterode am Harz, Germany). Briefly, the solution was frozen at −80 °C for 24 h. After freezing, frozen sample was placed in the freeze dryer at −45 °C for 5 days. The freeze-dried extract was stored at 4 °C in dark condition until further use. The extract yield of dried peels was expressed as mg of dried extract per g of dried peel matter.

### 2.5. GC-MS Analysis

Gas chromatography-electron ionization/mass spectrometry (GC-MS) analysis of phytochemical compounds present in RPE was carried out using Perkin Elmer Clarus 680 gas chromatography mass spectrophotometer (Agilent, Santa Clara, CA, USA). For sample preparation, RPE powder was dissolved in ethanol, centrifuged at 10,000 rpm for 10 min at 10 °C, and supernatant was used for GC-MS analysis. The GC-MS analysis was provided with an FID detector and Elite-5 capillary column ((5% biphenyl) 95% dimethylpolysiloxane), length 30 m × 0.25 mm ID and film thickness 250 µm df. Helium was used as carrier gas (flow rate, 1 mL/min). Injector and interface temperature was 260 °C. Column temperature was programmed from 60 to 300 °C at an increasing rate of 10 min, where it was held for 6 min. The spectrums of the compounds were compared with standard spectra available in Perkin Elmer GC-MS NIST library [14].

### 2.6. FT-IR Spectroscopy

The functional groups of compounds in RPE were identified by Fourier Transform Infrared Spectroscopy (FT-IR). A total of 10 mg of RPE powder was encapsulated in 100 mg of KBr pellet to prepare translucent sample disc. The powdered sample was then loaded in VERTEX 70 FT-IR spectrometer (Bruker Corporation, Leipzig, Germany), with a scan ranging from 500 to 3500 cm^−1^ and resolution of 4 cm^−1^ [14].

### 2.7. Minimum Inhibitory Concentration (MIC) and Minimum Bactericidal Concentration (MBC) Assays

The MIC of RPE was determined by broth microdilution method using 96-well microtiter plate recommended by Clinical and Laboratory Standard Institute (CLSI), 2015 [15]. The tested isolates were cultured in Mueller–Hinton broth (MHB) supplemented with (for *Vibrio* spp.) or without 1% NaCl for 4 h and adjusted to a concentration of 10^6^ cfu/mL with fresh MHB supplemented with or without 1% NaCl. Then, 100 µL of bacterial suspension was inoculated into 100 µL of RPE solution at the final concentrations, ranging from 32 to 16,384 µg/mL, and incubated at 30 °C for 18–20 h. After incubation, the MIC values were evaluated by adding 20 µL of 0.1% resazurin solution to each well and incubating them for 4 h at 30 °C. The lowest concentration of RPE that inhibited the growth of isolates was recorded as the MIC value. Bacteria were removed from each well that showed no growth, cultured on Tryptic Soy agar (TSA) supplemented with or without 1% NaCl, and incubated at 30 °C for 18–24 h. The lowest concentration showing no visible growth was taken as MBC value.

### 2.8. Antioxidant Activity Assays

2,2-diphenyl-1-picrylhydrazyl (DPPH) and 2,2′-azino-bis-(3-ethylbenzothiazoline-6-sulfonic acid) (ABTS) radical scavenging assays of RPE were examined in 96-well microtiter plate using the previous procedure described by Jitpakdee et al. [16].

#### 2.8.1. DPPH Assay

For the DPPH assay, 20 µL of RPE was added into 280 µL of freshly prepared 107.14 µM DPPH solution. Trolox was used as a standard control. The mixture was incubated in dark at room temperature for 30 min and the absorbance was measured at 517 nm with spectrophotometer (LUMIstar, BMGLABTECH, Ortenberg, Germany).

#### 2.8.2. ABTS Assay

The ABTS stock solution was prepared by adding 7 mM of ABTS with 8.75 mM of potassium persulfate and incubated in dark at room temperature for 16 h. Before use, the ABTS stock solution was diluted to 104.14 µM ABTS solution. Then, 20 µL of RPE or standard solution were mixed with 280 µL of 104.14 µM ABTS solution. After being mixed, the plate was incubated in dark at RT for 6 min and the absorbance was measured at 734 nm with spectrophotometer (LUMIstar, BMGLABTECH, Ortenberg, Germany).

#### 2.8.3. Scavenging Activity of RPE

The scavenging activity of RPE was expressed as the inhibitory concentration (IC_50_) which presents the concentration of the RPE required to inhibit 50% of DPPH and ABTS radicals. The experiments were done in three independent replicates. The % DPPH and ABTS scavenging activities were calculated using this formula:

[(control absorbance − sample absorbance)/control absorbance)] × 100

### 2.9. In Vitro Cytotoxicity of RPE

#### 2.9.1. Cell Line Culture

L929 (mouse, fibroblast, normal cell line), Vero (monkey, kidney, normal cell line), and MCF-7 (human, breast, adenocarcinoma) cells were purchased from ATCC (Manassas, VA, USA). L929 and MCF-7 were grown in RPMI 1640 (Invitrogen, Inchinnan, UK) supplemented with 10% fetal bovine serum (Invitrogen, Inchinnan, UK), 50 units/mL of penicillin (Invitrogen, Inchinnan, UK), and 50 µg/mL of streptomycin (Invitrogen, Inchinnan, UK). Vero cells were grown in DMEM (Invitrogen, Inchinnan, UK) supplemented with 50 units/mL of penicillin (Invitrogen, Inchinnan, UK) and 100 µg/mL of streptomycin (Invitrogen, Inchinnan, UK). Cells were incubated at 37 °C with 5% CO_2_ in a humidified incubator.

#### 2.9.2. Cytotoxicity Testing

The cytotoxicity testing of RPE on L929, MCF-7, and Vero cell lines took place using MTT [3-(4,5-dimethylthiazol-2-yl)-2,5-diphenyltetrazolium bromide] assay. L929, MCF-7, and Vero cell lines, at a density of 2 × 10^4^ cells, were incubated with RPE at concentration of 60–500 µg/mL in 96-well microtiter plate at 37 °C with 5% CO_2_ for 72 h. Then, cells were washed with 1× PBS and continued to incubate in 100 µL of 0.5 mg/mL MTT at 37 °C for 30 min. Avoiding light, the dark blue crystals of formazan (MTT metabolites) were dissolved with 100 µL of DMSO and incubated at 37 °C for 30 min. The absorbance at 570 and 650 nm were measured using microplate reader spectrophotometer [17]. The viable cells were presented as a percentage of survival and calculated using the following formula:

Survival (%) = [(A_570_ of sample − A_650_ of sample) / (A_570_ of control − A_650_ of control)] × 100

### 2.10. Statistical Analysis

Results were presented as the means ± standard deviations (S.D.) of three replicates. One-way analysis of variance (ANOVA) was used for comparison to more than two means. Correlations between DPPH and ABTS assays of antioxidant activity were calculated using Bonferroni post-test. A difference was considered statistically significant when *p* ≤ 0.05.

## 3. Results

### 3.1. GC-MS Analysis

The extraction yield of dried rambutan peels was 134.1 mg/g of dried matter. A total of 28 compounds in RPE were identified by the GC-MS analysis, representing 81.65% of the total components (Table S1). Ten major compounds (93.94 mg/g or 70.05% of the extraction yield) identified were mome inositol, catechol, 5-hydroxymethylfurfural, 2-pentenal, (E)-, acetic acid, 1,2,3-propanetriol, 2-furan-carboxaldehyde, 2-cyclopenten-1-one, 2-hydroxy-, 1,2-diphenylethan-1-ol, and phenol (Table 1).

### 3.2. FT-IR Spectroscopy

**Figure 1:**
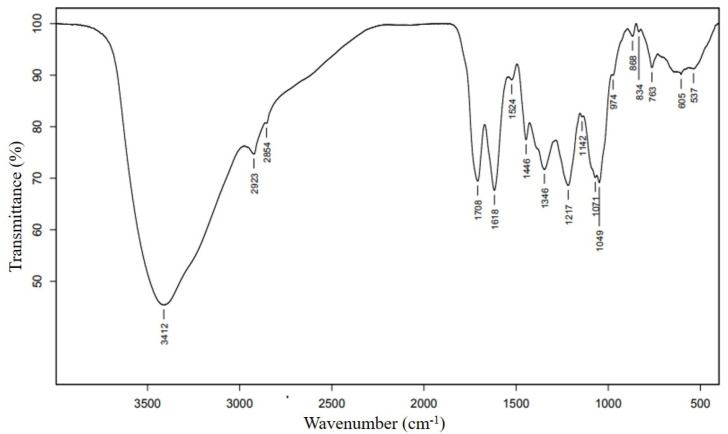
The FT-IR spectrum of rambutan peel extract (RPE).

The FT-IR spectrum was analyzed to identify the functional groups of bioactive compounds. The result represented the peak range of 537 to 3412 cm^−1^ (Figure 1) and FT-IR band assignments are indicated in Table 2. Multiple peaks found in this study represented the complex nature of RPE. A broad band peak at 3412 cm^−1^ indicated the presence of the O–H functional group. This strong absorption is because major compounds in RPE contain an O–H hydroxyl group in their structures, such as mome-inositol, catechol, 5-hydroxymethylfurfural, 2,3-propanetriol, -cyclopenten-1-one, 2-hydroxy-, and phenol. The band of 1217 and 1071 cm^−1^ confirmed the existence of a hydroxyl compound. Narrow bands at 2923 and 2854 cm^−1^ are characteristic peaks corresponding to the aliphatic C–H stretching vibration. In addition, the C–H bending mode was found in the fingerprint region of 1346 and 763 cm^−1^. The C=O stretching band at 1708 cm^−1^ indicated the presence of carbonyl compounds such as carboxyl, aldehhyde, ketone, or esters. This is in agreement with the presence of the 5-hydroxymethylfurfural, 2-pentenal, (E)-, acetic acid, 2-furan-carboxaldehyde, and 1,2-Diphenylethan-1-ol. The bands at 1618, 1142, and 1049 cm^−1^ indicated the presence of C–O stretching. The adsorption bands around 1524 and 1446 cm^−1^ were attributed to the aromatic rings. The bands at 974, 868, 834 indicated the carbohydrate fingerprint regions. Meanwhile, the band at 763 cm^−1^ corresponded to the =CH bending. In addition, no peak was found in the triple bond region (2000–2500 cm^−1^).

### 3.3. Antibacterial Activity of RPE against Food Pathogenic and Spoilage Bacteria

The antibacterial activity of RPE against nine bacterial strains was evaluated using MIC and MBC assays. The result showed that RPE could inhibited *B. subtilis*, *B. cereus*, and *S. aureus* at MIC values of 8192, 1024, and 2048 µg/mL, respectively. RPE could also inhibit the growth of *V cholerae* and *V. parahaemolyticus* with an MIC value of 2048 µg/mL. The MIC values were higher against *P. aeruginosa* and *P. fluorescens* (8192 µg/mL). However, RPE at the tested concentration was unable to inhibit *Salmonella* sp. PSU411 and *E. coli* ATCC25922. In this study, the MBC values were found to be varied. Regarding MBC/MIC ratios, RPE had a bactericidal effect (MIC/MBC ≤ 4) on *B. subtilis*, *B. cereus*, *V. cholerae*, *P. aeruginosa*, and *P. fluorescens* (Table 3).

### 3.4. Antioxidant Activities of the Extract

The antioxidant activity of RPE was determined by the DPPH and ABTS methods. The scavenging effect of RPE at the highest concentration (2048 µg/mL) against ABTS was significantly higher than DPPH radicals, with an IC_50_ of 494.25 ± 6.35 and 561.53 ± 6.30 µg/mL, respectively (Table 4).

### 3.5. Cytotoxicity of RPE

**Figure 2:**
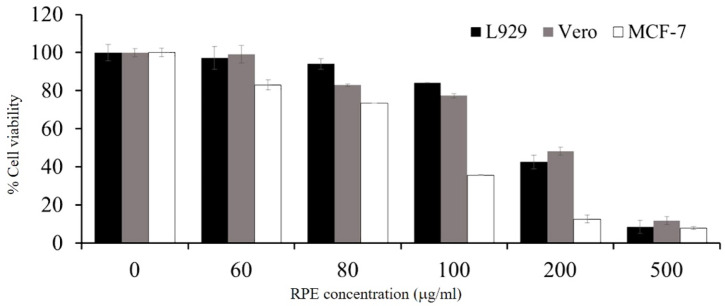
Cytotoxic activity of RPE on two normal cell lines (L929 and Vero) and one cancer cell line (MCF-7). Cell survival was measured by MTT assay. Data are shown as the mean ± standard deviation.

The cytotoxicity of RPE at different concentrations, ranging from 60 to 500 µg/mL, was tested against the proliferation of L929, Vero, and MCF-7 cell lines for 72 h. Cells were also evaluated by an MTT colorimetric assay. This study demonstrated that the growth inhibitory effect of RPE against each cell was dose-dependent. At the lowest concentration tested (60 µg/mL), RPE treatment resulted in cell viability of 97.17 ± 2.73% and 99.19 ± 0.46, for L929 and Vero normal cell lines, respectively. The cytotoxic effect of RPE on the MCF-7 cancer cell line (IC_50_; 94.11 ± 1.33 µg/mL) was stronger than that against L929 (IC_50_; 194.97 ± 4.87 µg/mL) and Vero (IC_50_; 205.92 ± 2.55 µg/mL) normal cell lines (Figure 2).

## 4. Discussion

The cultivation of Nasan School Rambutan (*N. lappaceum* L. cv. Rong Rian) in Thailand began in 1926, in the Nasan School, Bannasan, Surat Thani Province, Thailand. Then, its cultivation spread to other areas of Thailand. It is considered as the most delicious and famous rambutan cultivar grown in Thailand. Its shape is oval and average fruit weight is 40–50 g. When compared with See-chompoo cultivars, another popular rambutan cultivar grown in Thailand, cv. Rongrien has bigger seeds and a thinner aril than cv. See-chompoo [24]. Recently, various methods were used for the extraction of the bioactive compounds from its plants and agricultural wastes. Water extraction is of interest due to its natural, non-toxic, and environmental-friendly properties. The result of this study showed that the extract yield was high (134.1 mg/g). A previous study reported an observation of approximately 2.06 g/100 g of lyophilized rambutan peel [10]. The variation the in phytochemical components in the peels may be due to the cultivars and cultivation conditions, such as the soil condition, climate, and other physical factors [6]. Thitilertdecha and Rakariyatham (2011) reported that cv. Rongrien had a significantly higher number of phenolic compounds than that of the cv. See-compoo [25]. Unfortunately, most of the studies did not indicate the cultivar of rambutan in their publications. Thus, it is difficult to compare the similarity or differences in phytochemical compounds and bioactivities among difference cultivars.

This study investigated the phytochemical compounds present in the RPE extracted using water by GC-MS and FT-IR analyses. The results confirmed the presence of bioactive compounds in RPE. From FT-IR and GC-MS spectral analysis, it is clear that alcoholic and phenolic compounds are present in RPE. A previous study showed that different parts of rambutan or solvents affect the presence of the extracted bioactive compounds. Aziz et al. reported that the extract from rambutan seeds in Malaysia contains the functional group of O–H, N–H, and C=O [26]. Rakariyatham et al. described how the water extraction of rambutan peel showed p-Coumaroyl glucose, vanillic acid, hexoside, carboxylic acid, ellagic acid, corilagin, and geraniin. Moreover, ethyl gallate and geraniin were obtained by ethanol extraction. The major compounds identified in the methanolic extract of rambutan peel were ellagic acid, corilagin, and geraniin [27]. Monrroy et al. (2020) reported 18 active compounds in RPE which had not previously been characterized [18]. However, some compounds, including catechol or 1,2-benzenediol (Table 1) and 5-methylfuran-2-carbaldehyde (Table S1), were also identified in this study.

In this study, nearly 50% of the major compounds were mome inositol and catechol which contain the -OH group. Mome inositol is a phytochemical compound found in several medicinal plants such as *Zilla spinosa* (Forssk.), *Corbichonia decumbens* (Forssk.), and *Securigera securidaca* (L.). It acts as an anti-alopecic, anti-cirrhotic, anti-neuropathic, cholesterolytic, lipotropic, and sweetening property [28]. Mome inositol was found to have anti-proliferation, anti-alopecic, anti-cirrhotic, and anti-neuropathic activities [13,29]. In addition, the catechol compound was responsible for antifungal, antibacterial, and antioxidant activities [30,31]. The content of catechol obtained in this study was 29.37 mg/g of dry rambutan peel. This was higher than a previous study with sweet orange, lemon, tangerine, and grapefruit extracted by water (range between 37.00 to 52.16 mg catechol/100g dry weight) [32].

The 5-hydroxymethylfurfural, acetic acid, and 2-furan-carboxaldehyde reported in this study were similar to the major organic compounds of *Jatropha curcas* L. kernel meal extract which exhibited antioxidant and antibacterial activities [33]. The antibacterial, antioxidant, and various biological activities of these compounds are widely reported in many fruit peels. For example, the 5-hydroxymethylfurfural from banana (*Musa acuminata*) peels possess antibiofilm and antivirulence against *Pseudomonas aeruginosa* [34]. The identification of 5-hydroxymethylfurfural was reported in the hydromethanolic extract of pomegranate (*Punica granatum* L. var. *nana*) peel and the extract exhibited antimicrobial activity against various plant pathogens [35]. 5-hydroxymethylfurfural and 2-furancarboxaldehyde were reported as two major compounds present in mango (*Mangifera indica* L.) peels and other plants, and their antioxidant and antiproliferative potential have been reported [36,37]. Acetic acid has been reported as an antimicrobial agent used for disinfection for more than 6000 years [38]. The presence of acetic acid, antioxidant properties, and antimicrobial activity has also been reported in banana peels [39]. The presence of phenol, unsaturated aldehydes, and ketones have been reported in many fruit peels, including melon, watermelon, orange, and mango [40,41]. The presence of these compounds contributes to fruit flavor and supports the antioxidant properties [42].

Previously, the bioactivities of RPE, such as anticancer, antiviral, antidiabetic, and anti-hypercholesterolemic activities, were reported [40]. In the present study, we investigated the antibacterial activity of RPE against three Gram-positive and six Gram-negative bacteria. The result showed that RPE could inhibit various pathogens, except *Salmonella* sp. and *E. coli*. Phuong et al. also examined the effect of RPE on the growth of bacterial pathogens. RPE at 1000 µg GAE/mL affected the growth of *Vibrio* spp. (including *V. campbellii*, *V. parahaemolyticus*, and *V. anguillarum*) and *S. aureus*. However, RPE had no effect on *E. coli* growth [5]. Tadtong et al. also reported that RPE at concentration of 2 mg/mL could inhibit *S. aureus*, but had no ability to inhibit *E. coli* by a disc diffusion assay. The results showed that flavonoids and tannins (members of phenolic compounds) were the major bioactive compounds [4]. In addition, Tsong et al. described that RPE could inhibit both Gram-positive and Gram-negative bacteria at a concentration of 0.5 mg/mL for *S. typhi*, *B. cereus*, and *B. subtilis*, and 1 mg/mL for *E. coli* [12]. In the study of Thitilertdecha et al., *V. cholerae* and *S. aureus* was inhibited by RPE at an MIC of 15.6 mg/mL and 31.2 mg/mL, respectively [10]. The antibacterial activity of RPE depends on the amount of bioactive and phenolics compounds present in the peel, such as geraniin, ellagic acid, rutin, quercetin, corilagin, flavonoid, tannin, and triterpenoid [5,12]. Moreover, bacterial cell structures may act as the important factors for bacteria which are resistant against antimicrobial agents [43]. This study demonstrated the potential of RPE as an antibacterial agent against important food pathogenic bacteria, especially *B. cereus* and *V. cholerae*.

In this study, the ability to scavenge the free radicals of RPE was determined by DPPH and ABTS assays. Both methods were widely used to examine the antioxidant activity of the extract, based on the ability of proton radical scavenging or hydrogen donating of the extract [44]. The DPPH and ABTS assays are convenient methods recommended for the primary screening of the antioxidant activity in plants and essential oils [45,46]. However, the disadvantage of these methods has been reported, especially the length of incubation time and the solution preparation [47]. In our study, the DPPH and ABTS radical scavenging activities of RPE were significantly different (*p* < 0.05). The difference may be due to the position of the aromatic ring of the hydroxyl group influencing the quenching of free radicals [48]. In a previous study, the free phenolic compound of RPE, obtained by microwave-assisted extraction, showed an IC_50_ of 3.55 ± 0.32 µg/mL by the DPPH assay [49]. Thitilertdecha et al. also reported the antioxidant activity of major phenolic compounds from the methanolic extract of rambutan peel, including of geraniin, corilagin, and ellagic acid, for which the IC_50_ were 0.79, 1.42, and 1.64 µM, respectively [50]. Previous studies reported that ellagitannin, phenolic acid, quercetin, and gallotannin were responsible for the antioxidant activity of RPE [10,44]. The differences in antioxidant activity may be due to the amount of antioxidant substances present in the extract, such as polyphenol, vitamin C and E, and carotenoids [51]. Thus, further study of the antioxidant activity of the purified active compounds is needed.

To evaluate the toxic effect of RPE, we also investigated the cytotoxic activity against various cell lines, including L929, Vero, and MCF-7 using an MTT assay. There are no previous data showing the toxicity levels against these cell lines. Our results showed that crude RPE showed a lower toxicity on L929 and Vero cell lines compared to MCF-7. Okonogi et al. evaluated the cytotoxicity of RPE on human colon adenoma (Caco-2) and normal peripheral blood mononuclear (PBMC) cell lines. The results showed that RPE exhibited non-toxic properties on a normal cell line with high IC_50_ values (>100 μg/mL) [52]. According to the standard National Cancer Institute (NCI) criteria and Geran protocol [53], the author also suggested that RPE is non-toxic to normal human cells and has the potential to develop as a cancer-preventive agent. Hernandez-Hernandez et al. revealed that the methanolic extract of yellow rambutan peel extract showed a cytotoxic effect on breast cancer cells (MDA-MB-231) and osteosarcoma cancer (MG-63) cell lines at a very low concentration (IC_50_ 5.42 ± 1.67 and 6.97 ± 1.02 μg/mL, respectively), whereas no effect on the cervical cancer (HeLa) cell line was observed [54]. Our study demonstrated that RPE at 100 μg/mL could inhibit more than 50% of MCF-7, while 84.12 ± 3.59 and 77.41 ± 2.05% cell viability was observed in L929 and Vero cell lines, respectively.

Overall, the present study demonstrated that RPE obtained by the water extraction method contained phytochemical compounds with good bioactivities and a low toxicity. The characterization of the pure active compound is, therefore, attractive for further utilization and development of value-added products.

## 5. Conclusions

The results obtained in this study provided valuable information about the phytochemical composition and bioactivities of “Rong Rian” rambutan peel extract, including its antibacterial, antioxidant, and cytotoxic properties. This extract should, therefore, be used in many applications, such as in natural anti-microbial agents in the food industry or as a natural food preservative.

## Figures and Tables

**Table 1:** Ten major organic compounds identified in rambutan peel extract (RPE).

No.	Name of Compound	Formula	Amount (mg/g of Dry Peel)	Retention Time	Match Factor
1	Mome Inositol	C_7_H_14_O_6_	35.99	56.7432	87.8
2	Catechol (1,2-benzenediol)	C_6_H_6_O_2_	29.37	45.6479	96.1
3	5-Hydroxymethylfurfural	C_6_H_6_O_3_	5.69	41.5651	94.9
4	2-Pentenal, (E)-	C_5_H_8_O	5.22	20.9707	87.4
5	Acetic acid	C_2_H_4_O_2_	3.69	15.5889	99.0
6	1,2,3-Propanetriol	C_3_H_8_O_3_	3.67	37.5876	96.2
7	2-furan-carboxaldehyde	C_5_H_4_O_2_	2.66	15.7927	96.1
8	2-Cyclopenten-1-one, 2-hydroxy-	C_5_H_6_O_2_	2.63	24.5133	96.0
9	1,2-Diphenylethan-1-ol	C_14_H_14_O	2.63	40.2940	97.2
10	Phenol	C_6_H_6_O	2.40	30.6622	95.2

**Table 2:** Peak position (cm^−^^1^) and tentative assignments of FT-IR absorbance bands for RPE recorded in the spectral region from 500 to 3500 cm^−^^1^.

Peak Position (cm^−1^)	Tentative Assignment	Reference
3412	O–H stretching (hydroxyl)	[18,19]
2923, 2854	C–H stretching (alkyl)	[18,19]
1708	C=O stretching (carboxyl, aldehyde, ketone, ester)	[18,19]
1618	C–O stretching	[20]
1524	C–C (aromatic ring)	[21]
1446	C–C (aromatic ring)	[18]
1346	C–H bending (alkyl)	[18,20,22]
1217	O–H bending (hydroxyl)	[18,19]
1142	C–O stretching	[21]
1071	O–H bending (hydroxyl)	[19]
1049	C–O stretching	[18]
974	Carbohydrate	[23]
868	Glycosidic linkage	[19]
834	Carbohydrate	[19,23]
763	=CH bending	[18]
605	Unknown	
537	Unknown	

**Table 3:** The MIC and MBC values of rambutan peel extract (RPE) against various food pathogenic and spoilage bacteria.

Bacteria	MIC (µg/mL)	MBC (µg/mL)	MBC/MIC Ratio
Gram-positive bacteria			
*Bacillus subtilis* PSU4655	8192	16,384	2
*Bacillus cereus* PSU3874	1024	2048	2
*Staphylococcus aureus* ATCC25923	2048	>16,384	ND
Gram-negative bacteria			
*Vibrio cholerae* PSU6072	2048	8192	4
*Vibrio parahaemolyticus* ATCC17802	2048	>16,384	ND
*Salmonella* sp. PSU411	>16,384	>16,384	ND
*Escherichia coli* ATCC25922	>16,384	>16,384	ND
*Pseudomonas aeruginosa* ATCC27853	8192	16,384	2
*Pseudomonas fluorescens* PSU6074	8192	16,384	2

ND: not determined.

**Table 4:** Antioxidant activity of rambutan peel extract (RPE).

Samples	Antioxidant Activity	
	DPPH IC_50_ ^a^ (µg/mL)	ABTS IC_50_ (µg/mL)
RPE	561.53 ± 6.30 *	494.25 ± 6.35 *
Trolox	30.38 ± 0.56	27.37 ± 0.15

^a^ IC_50_ is the 50% inhibitory concentration. The value indicates the mean ± SD for three independent experiments performed in triplicates; * *p* < 0.05 compared between DPPH and ABTS.

## Data Availability

Data sharing is not applicable to this article.

## Supplementary Materials

The following supporting information can be downloaded at: https://www.mdpi.com/article/10.3390/antiox11050956/s1, Table S1: Summary of phytochemical compounds in rambutan peel extract (RPE) by GC-MS.
