# Supplementary material for: Phytochemical Composition and Bioactivities of Aqueous Extract of Rambutan (Nephelium lappaceum L. cv. Rong Rian) Peel
Source: Antioxidants (Basel). 2022 May 12;11(5):956. doi: 10.3390/antiox11050956 (PMC9137458; doi:10.3390/antiox11050956)
Supplement: Supplementary file 1 [file antioxidants-11-00956-s001.zip › antioxidants-1696303-supplementary.pdf]

Table S1. Summary of phytochemical compounds in rambutan peel extract (RPE) by GC-MS.

RT	Compound name	CAS#	Formula	Component area	Match factor	% of Total
10.1546	2-(2-Methylphenyl)ethanol	19819-98-8	$C_9H_{12}O$	40407	82.9	0.87
10.5000	1-Hexen-1-one	89723-54-6	$C_6H_{10}O$	11376.5	90.1	0.22
15.5889	Acetic acid	64-19-7	$C_2H_4O_2$	210822.5	99	2.75
15.7927	2-Furan-carboxaldehyde	98-01-1	$C_5H_4O_2$	87888.4	96.1	1.98
17.9283	Butanoic acid, 2-hydroxy-, methyl ester	29674-47-3	$C_5H_{10}O_3$	11178.7	86.6	0.28
19.0076	5-Methylfuran-2-carbaldehyde	620-02-0	$C_6H_6O_2$	20363.8	86.9	0.24
19.1055	2-Butanol, 3-methyl-, (S)-	1517-66-4	$C_5H_{12}O$	14680.6	82.9	0.02
20.9707	2-Pentenal, (E)-	1576-87-0	C_5H_8O	249447	87.4	3.89
21.5688	3-Furanmethanol	4412-91-3	$C_5H_6O_2$	23184.6	89.9	0.51
21.7451	Myrcene	123-35-3	$C_{10}H_{16}$	10805.9	88.9	0.38
24.5133	2-Cyclopenten-1-one, 2-hydroxy-	10493-98-8	$C_5H_6O_2$	113223.4	96	1.96
27.1529	Cellulose pyrolysis product	2000020-96-8	$C_5H_6O_3$	17479.1	85.8	0.36
28.9044	2-Propenamide	79-06-1	C_3H_5NO	21029.7	88	0.42
30.0091	Levoglucosenone	37112-31-5	$C_6H_6O_3$	48424.4	83.5	0.86
30.3314	" 5,6 - dihydro - pyran - 2,5 - di - one " (so Pastorova) questionable name	5926-95-4	$C_5H_4O_3$	51795.3	97.1	0.95
30.6622	Phenol (hydroxybenzene)	108-95-2	C_6H_6O	102451.9	95.2	1.79
31.7726	2H-Pyran-2-one, 4-hydroxy-6-methyl-	675-10-5	$C_6H_6O_3$	11691	80.8	0.50
32.4191	Dihydroxyacetone	96-26-4	$C_3H_6O_3$	124683.2	90.2	1.76
33.2447	2,5 and 2,6-dimethyl dioxene	2000021-48-0	$C_6H_{10}O_2$	38421.6	85	0.71
34.4638	3-Furanol, tetrahydro-	453-20-3	$C_4H_8O_2$	13540.2	80.5	0.45
36.6134	2,3-Dihydro-3,5-dihydroxy-6-methyl-4H-pyran-4-one	28564-83-2	$C_6H_8O_4$	94024.3	95.4	1.64
37.5876	1,2,3-Propanetriol (glycerol)	56-81-5	$C_3H_8O_3$	237905.7	96.2	2.74
40.294	1,2-Diphenylethan-1-ol	2000196-91-3	$C_{14}H_{14}O$	128537	97.2	1.96
41.3781	Decanoic acid, ethyl ester	110-38-3	$C_{12}H_{24}O_2$	16216.6	89.1	0.58
41.5651	5-Hydroxymethylfurfural	67-47-0	$C_6H_6O_3$	278644.4	94.9	4.24
45.6479	Catechol (1,2-benzenediol)	120-80-9	$C_6H_6O_2$	1316339.3	96.1	21.90
48.8704	Propane, 2-methoxy-2-methyl-	1634-04-4	$C_5H_{12}O$	42148.8	82.2	0.85
56.7432	Mome inositol	0-00-0	$C_7H_{14}O_6$	3878135.5	87.8	26.84
